# Effects of Crowding and Water Flow on Golden Shiners *Notemigonus crysoleucas,* Held in a Flow Tank

**DOI:** 10.3389/fphys.2022.875898

**Published:** 2022-04-29

**Authors:** Sindhu Kaimal, A. H. Haukenes, Nilima N. Renukdas, Anita M. Kelly

**Affiliations:** ^1^ Department of Aquaculture and Fisheries, The University of Arkansas at Pine Bluff, Pine Bluff, AR, United States; ^2^ Zuni Public School District, Zuni, NM, United States; ^3^ Washington Department of Fish and Wildlife- Science Division, Olympia, WA, United States; ^4^ Arkansas Department of Agriculture, Aquaculture Division, Little Rock, AR, United States; ^5^ School of Fisheries, Aquaculture and Aquatic Sciences, Alabama Fish Farming Center, Auburn University, Greensboro, AL, United States

**Keywords:** stress, cortisol, crowding, split-pond, growth

## Abstract

Split-pond systems (SPS) such as those used for catfish are being considered for raising baitfish. When using these systems for baitfish such as golden shiners *Notemigonus crysoleucas*, an important factor to be considered is how well the species can tolerate crowding, as the design mandates fish be confined to a smaller portion of the pond. Another aspect of the SPS design is the water flow between the two units for at least 10–14 h each day. SPS can be successfully implemented for other species if factors such as crowding, and water flow do not affect growth. Two laboratory studies were conducted each using 12, 40-L tank flow-through system to observe the growth and physiological performance of golden shiners held for 28 days at three crowding densities: 600; 1,200, and 2,400 fish/m^3^, keeping water flow at 1 cm/s (Experiment 1) and using three flow rates: 1, 2, and 4 cm/s at similar densities (600 fish/m^3^) (Experiment 2). At the end of the experiments, fish were subject to acute 1-min confinement stress and whole-body cortisol was measured at 30 min intervals for up to 2 h to monitor the secretion pattern and recovery. Results from experiments showed no difference in the final weight, length, feed conversion, and survival among treatment groups; survival rates were lower in the flow study because of columnaris *Flavobacterium covae* infections. Baseline cortisol was also not different among the treatments. Cortisol increased 30 min after the acute stress and returned to near baseline in 2 h in the crowding study suggesting acclimation to the chronic stressor. However, in the flow study, cortisol remained elevated even after 2 h, and hence a compromised pathophysiological response. Crowding and water flow do not impair feed intake, growth, or survival in golden shiners, and in these aspects may be a suitable species for SPS.

## 1 Introduction

In the southern U.S., baitfish are raised in traditional earthen ponds (TEP). Recently, interest in split-pond systems (SPS) for raising baitfish, such as golden shiners *Notemigonus crysoleucas*, has increased among Arkansas producers after the success achieved in hybrid catfish (*Ictalurus punctatus* ♀ X *I. furcatus* ♂) production with the same systems. However, the biological and physiological effects of raising baitfish in SPS are unknown. The SPS designed primarily for hybrid catfish are constructed by dividing an existing TEP in a 1:4 ratio. Fish are confined to the smaller portion of the pond, the fish culture unit (FCU), while the remaining portion is used as a natural waste-treatment unit (WTU). Oxygen-rich water is allowed to circulate between the two units of the pond during the day by a pump or a paddlewheel. At night, when photosynthesis stops, the oxygen levels drop, and aerators are turned on in the FCU. This SPS design, separating the FCU from the WTU, addresses some inefficiencies of TEP by allowing a natural augmentation of water quality by photosynthesis and chemosynthesis (Farrelly et al., 2015), thereby facilitating increased fish production.

As production techniques in aquaculture evolve from extensive to more intensive, culture conditions for fish tend to become more stressful. While exposure to sublethal stressors causes compromised biological functions such as poor growth and poor immunity leading to an increase in diseases incidences, lethal stressors often result in mortality. It is therefore imperative to identify and manage stressors for successful aquaculture practices. Water quality, culture procedures (crowding, handling, and transportation), and biological interactions among fish are the most detrimental stressors in intensive fish culture (Wedemeyer 1997). Fish loading density determined by the carrying capacity of the culture system is limited by water quality variables and fish metabolites while crowding in terms of a “behavioral necessity for physical space” is species-specific.

The severity and duration of exposure to the stressor determine stress effects which may be manifested at the cellular level through to the entire population. At the cellular level, primary responses to stress in fish include increased catecholamines, corticosteroids, and cortisol. Plasma cortisol helps maintain hyperglycemia after the effects induced by catecholamines have subsided (Barton and Iwama 1991). If the stress is severe, secondary responses are triggered causing changes in blood biochemistry including increases in plasma glucose, hematocrit, lactate, heart and metabolic rate, and gill permeability, and decreases in sodium, glycogen, plasma chloride, potassium, muscle protein, and hydromineral balance concentrations. Exposure to chronic stresses causes tertiary responses such as a decrease in growth, disease resistance, swimming capability, feeding, reproductive capacity, and survival (Barton and Iwama 1991; Wedemeyer 1997; Portz et al., 2006).

Using SPS for intensive aquaculture has shown promise for increasing catfish production; producers are now encouraged to expand SPS use for other food fish and baitfish species. While water quality variables will most certainly not be an issue impacting growth in a SPS, crowding fish is likely to become a stressor. Also, in typical aquaculture practices, baitfish are not generally exposed to flowing water. In SPS water is circulated between the FCU and the WTU for part of the day, which may act as a stressor for fish that are not adapted for a life in flowing water. Two laboratory studies were conducted under fixed environmental conditions to evaluate the effects of stressors, crowding and water flow, on golden shiners physiology and growth.

## 2 Methods

Two 28-day experiments were conducted to determine 1) the effect of crowding and 2) the effect of flow rate on golden shiner growth and physiology. These studies were conducted at the Hatchery Research and Demonstration (HRD) laboratory, Aquaculture Research Station, University of Arkansas at Pine Bluff. Both studies were conducted with a common experimental setup and identical methods.

### 2.1 Experimental Flow Tanks

Fish were tested in partially recirculating flow tanks (after Vogel and LaBarbera 1978). Twelve 14 L flow tanks (300.00 cm × 7.50 cm x 6.25 cm) constructed from polyvinyl chloride (PVC) gutters were used for the experiments. A 500 L head tank (30.5 × 55.9 × 365.76 cm; Red Ewald, Karnes City, Texas) was used to supply water to the 12 flow tanks; the flow to each tank was regulated by a gate valve. Water from each flow tank exited through an elbow into a 1500-L sump. The flow rate was calibrated by determining the volume leaving each tank over time and converting this data to flow rate using the dimensions of the flow tank according to the equation:

Velocity =(4∗flow rate)/(π∗diameter^ 2)

**FIGURE 1:**
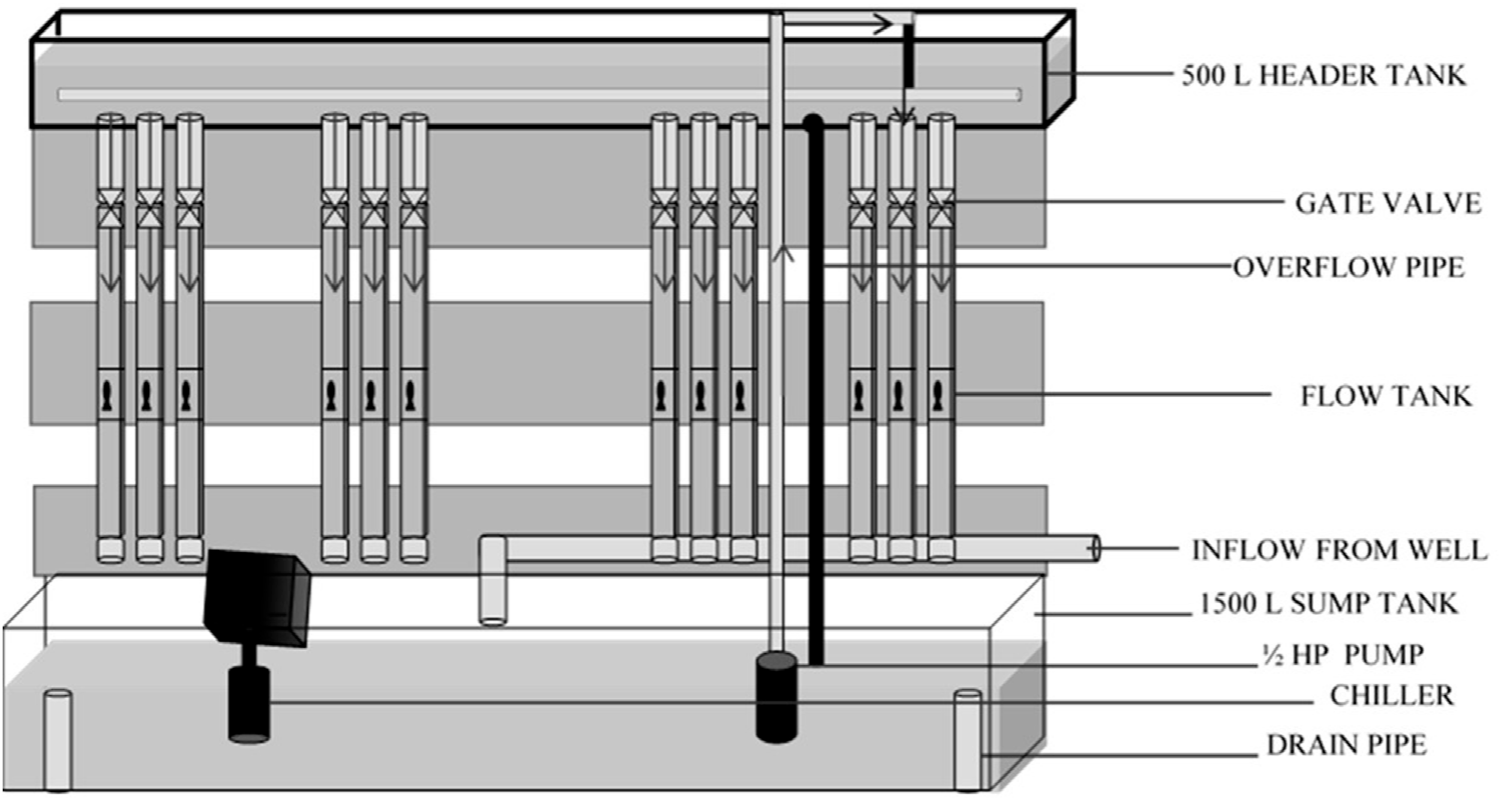
Schematic of experimental set-up.

**FIGURE 2:**
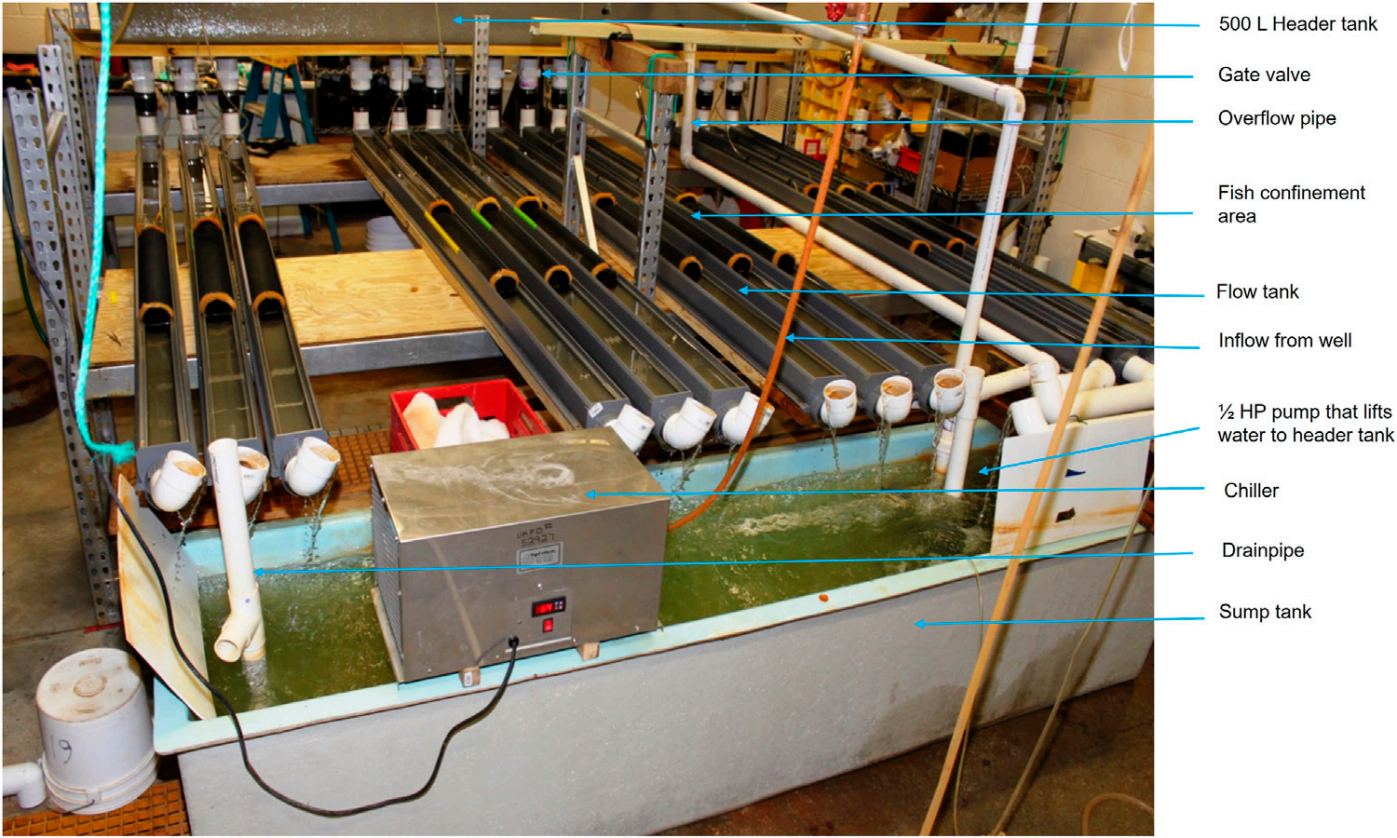
Photograph of actual experimental set-up. Fish were confined within the flow tanks by screens made of polypropylene honeycomb. The tanks were covered with a plastic screen mesh to prevent the fish from jumping out.

A 0.37-kW (½ HP) submersible sump pump (Dayton Pumps, Dayton Water Systems, West Carrolton, Ohio) was used to re-lift water to the head tank and mixed with a constant inflow of fresh well water to prevent waste product accumulation. A 0.75-kW, drop-in chiller (Frigid Units Inc., Toledo, Ohio) was used to maintain a constant water temperature (∼21°C) and remove heat produced by the pump (Figure 1 and Figure 2). Screens made of polypropylene honeycomb (Plascore, Zeeland, Michigan) were used to remove turbulence from the flow and confine fish within an experimental zone. The lab was illuminated with artificial lighting and set on a 12:00-h light: 12:00-h dark cycle. The temperature of ambient air in the laboratory was maintained at ∼ 21°C. Air stones were used to provide supplemental aeration in each flow tank to maintain dissolved oxygen (DO) concentrations ≥5 mg/L.

### 2.2 Fish

Juvenile golden shiners weighing about 1 ± 0.38 g were obtained from a commercial farm in Lonoke, Arkansas. The fish were inspected at the disease diagnostic laboratory at the University of Arkansas at Pine Bluff and were found to be free of any clinical signs of disease before the experiments. A 700-L recirculating tank (Living Stream system, Frigid Units, Toledo, Ohio) was used to acclimate fish to the laboratory for 5 days and to hold extra fish for Experiment 2. During acclimation, fish were fed a commercial diet once daily at 1% body weight.

Fish were stocked into each flow tank for both experiments. The tanks were covered with a plastic screen mesh to prevent the fish from jumping out (Figure 2). Within the first 5 days of each experiment, dead fish were replaced. Fish were fed a 1.5-mm pellet commercial diet once daily to apparent satiation (45% crude protein and 16% crude fat; Skretting, Toole, Utah). Fish were fed by hand in small amounts to satiation; feed was weighed in vials before and after feeding to calculate daily amounts.

During the experiments, dead fish were removed and, the mesh barriers were adjusted to confine fish to their original densities; consequently, “crowding” effects did not vary during the study. Dead fish were examined for external signs of disease, such as *Flavobacterium covae*. As dead fish are not normally used to test for bacteriological assays, and the live fish were still under experimental conditions, live fish samples for bacterial culture were collected only after the experiments were complete.

Two fish per treatment were tested to check for *Flavobacterium covae*. Fish were euthanized in MS222 and then placed in 70% ethanol to disinfect the surface before incisions were made to expose the organs. The samples were streaked on Brain Heart Infusion agar and Hsu-Shots agar plates. The bacterial plates were then placed at room temperature (∼20°C) for 48 h after which they were examined for bacterial growth.

### 2.3 Water Quality

Total hardness, total alkalinity, and chlorides were measured at the start of the study. Other water quality variables (DO, pH, and total ammonia nitrogen (TAN)) were measured daily from water within the header tank and sump. DO was measured with a DO meter (Hach Co., Loveland, Colorado), pH was measured with a pH meter (Denver Instrument Model UP-5), and TAN was measured by the salicylate method (Hach Co., Loveland, Colorado). Total alkalinity and total hardness were measured with Hach digital titration kits (APHA 1992).

### 2.4 Exposure to Chronic Stressors

#### 2.4.1 Experiment One: Effect of Crowding

Effects of density or crowding on fish growth and physiology were evaluated in these experiments. Thirty fish were held in each flow tank under three density treatments: 600, 1,200 or 2,400 fish/m^3^ with four replicates per treatment (*N* = 12). These densities were used to simulate a typical stocking rate of 375,000 fish/ha (Stone et al., 2016), double the stocking rate and triple the stocking rate. Screens were used to confine fish to limited areas within the flow tank. Water flow velocity was maintained at 1.0 cm/s in all the tanks.

#### 2.4.2 Experiment Two: Effect of Flow Rate

Effects of flow rate on fish growth and physiology were evaluated in this experiment. Thirty-five fish were held at 600 fish/m^3^, and treatments consisted of three flow rates: 1 (or 2.7 L/min), 2 (or 5.5 L/min), or 4 (or 10.9 L/min) cm/s, with four replicates per treatment (*N* = 12). Water flow rate at 1 cm/s was used to simulate the lentic environment as a no-flow treatment would have compromised water quality. The 2 cm/s was used to simulate the water flow in an experimental SPS in which flow into the FCU occurs at 3.7 m^3^/min over an area of 3.35 m^2^ which is about 2 cm/s (Smith and Stone 2017). Finally, the 4 cm/s was double the flow rate of the experimental SPS used in previous studies (Smith and Stone 2017).

#### 2.4.3 Harvest

At the completion of each 28-day study, two or 5 fish per tank across all treatment groups were removed carefully without disturbing the remaining fish just before conducting the acute handling experiment. The fish were immediately euthanized in a 400 mg/L dose of tricaine methanesulfonate (MS222) to be used as baseline (0:00-h) measurements of cortisol.

#### 2.4.4 Acute Handling Challenge Stressor

The remaining fish in each flow tank was subjected to an acute handling challenge by confining the fish further to replicate the net-stress challenge for 1 min. The fish were then placed back into the original densities. Five fish from each treatment group were sampled 0:30 h; 1:00 h; 1:30 h and 2:00 h after the simulated net-stress.

#### 2.4.5 Growth and Physiology Measurements

Weight and length were measured for all fish, which were stored on ice and then frozen at −80°C until analyzed for cortisol. Survival rates were recorded across all treatment groups and replicates. Feed conversion ratio (FCR) was determined from consumption and weight gain measurements.

FCR=feed consumed (g)/weight gained (g)

#### 2.4.6 Cortisol Analysis

Whole-body cortisol extraction was performed as described by Sink et al. (2007). Briefly, a pooled sample of 2-5 fish from each tank and time series group were homogenized using a Stomacher ^®^ 80 laboratory blender (Seward Ltd., Norfolk, United Kingdom). For cortisol extraction, 2 mL of PBS (0.26 g of K_2_HPO_4_, 2.17 g of Na_2_HPO_4_- 7H_2_O and 8.71 g of Na Cl adjusted to 1 L with deionized water at pH 7.4), 100 µL of vegetable oil/g of body weight and 4 mL of diethyl ether were added to the homogenized sample. The sample was mixed thoroughly, vortexed for 1 min, and then centrifuged at 4,000 *× g* for 5 min. After centrifugation, the test tubes were immediately frozen at −20°C overnight. The unfrozen portion (diethyl ether containing cortisol) was decanted into a weighed 10-mL test tube. The extract was evaporated under a gentle stream of nitrogen for 2 h to obtain a lipid extract containing cortisol. Cortisol concentration was determined from the extract with a previously validated cortisol enzyme-linked immunosorbent assay (ELISA) (Assay Designs Inc., Kit 900–071; Sink et al., 2007).

#### 2.4.7 Statistical Analysis

Data were analyzed with SAS ver 9.4 (SAS Institute Inc., Cary, North Carolina). Growth (weight and length gain), survival, and FCR were compared across all treatment (N = 12) groups for both experiments using a one-way analysis of variance (ANOVA). Cortisol concentration at various time intervals was compared across all treatment groups using a two-way ANOVA repeated measures design. Cortisol data were log transformed to meet the assumptions of normality and equal variance. Percent data were arcsine square-root transformed. Significance was assessed at *p* ≤ 0.05. If significant differences were found, Tukey’s LSD post hoc test was used to determine where significant differences occurred.

## 3 Results

### 3.1 Water Quality

During both 28 day studies, DO concentration (9.2 (±0.3) mg/L), TAN (0.06 (±0.03) mg/L) and pH (7.07 (±0.19)) were maintained at optimum levels. Temperature in the header tank and sump was maintained at 20.85 (±1.44)°C.

### 3.2 Experiment 1: Crowding Treatments

At the end of the study, mean (±SD) golden shiner weights held at densities of 600, 1,200, or 2,400 fish/m^3^ were 1.12 (±0.05), 1.08 (±0.08), and 1.12 (±0.12) g, respectively, which did not vary significantly among the treatments. The observed average FCR ranged between 2.22 and 2.36, while mean survival was between 91 and 98%, both of which also did not vary among treatments (Table 1). On average, fish in the three treatment groups gained 20% of the initial body weight (0.95 g) during the 28-days period.

**TABLE 1:** Survival, weight, feed conversion ratios (FCR) and whole-body cortisol concentrations for golden shiners held at various densities and subject to constant flow rates or held at same densities but subjected to various flow rates.

Variables	Crowding Experiment				Flow-rate Experiment					
	600 Fish/m^3^	1,200 Fish/m^3^	2,400 Fish/m^3^	Comparison		1 cm/s	2 cm/s	3 cm/s	Comparison	
				*F*_(2, 9)_	*p-*value				*F*_(2, 9)_	*p-*value
Survival (%)	97.5 ± 3.19	94.17 ± 3.19	90.83 ± 6.87	1.97	0.1948	48.57 ± 15.99	68.57 ± 21.00	47.14 ± 4.95	2.39	0.1472
Weight (g)	1.12 ± 0.05	1.08 ± 0.08	1.12 ± 0.13	0.29	0.7564	1.85 ± 0.10	1.73 ± 0.24	2.07 ± 0.27	2.45	0.1418
FCR	2.24 ± 0.18	2.36 ± 0.03	2.22 ± 0.12	1.35	0.3061	1.68 ± 0.10	1.77 ± 0.22	1.52 ± 0.21	1.82	0.2015
Cortisol level (ng/g)										
Baseline	10.41 ± 13.34	3.42 ± 2.88	9.02 ± 11.73	0.62	0.5592	4.10 ± 5.57	2.62 ± 2.12	5.07 ± 6.57	0.09	0.9112
0.5 h	16.55 ± 10.28	57.48 ± 94.28	30.66 ± 9.51	0.45	0.6487	35.61 ± 41.05	62.95 ± 69.31	28.18 ± 19.93	0.14	0.8754
1.0 h	15.80 ± 15.86	35.41 ± 33.09	4.03 ± 1.49	2.97	0.1020	52.14 ± 22.65	24.07 ± 17.13	69.90 ± 34.97	3.78	0.0644
1.5 h	23.67 ± 14.50	24.55 ± 13.57	11.33 ± 11.10	2.53	0.1339	54.71 ± 35.79	46.73 ± 31.15	7.76 ± 10.81	3.84	0.0624
2.0 h	18.91^a^ ± 8.42	9.93^ab^ ± 3.74	5.74^b^ ± 2.16	4.95	0.0355	74.11 ± 55.05	69.78 ± 55.11	71.31 ± 39.43	<0.01	0.9992

Measurements of whole-body cortisol were significantly different between the 600 and 1,200 fish/m^3^ at the 2 h interval measurement. The whole-body cortisol concentrations in the 2,400 fish/m^3^ were significantly lower than those of the 600 fish/m^3^ (Table 1). Cortisol concentrations also varied significantly across sampling time intervals (F_4, 60_ = 4.85; *p* = 0.002). Baseline cortisol was on average 7.6 (± 9.9) ng/g body weight which was significantly lower than measurements at other time intervals. Cortisol significantly increased 30 min after the acute stress in all the treatment groups to an average of 34.9 (±20.8) ng/g. The concentrations dropped during the next hour to an average of 19 (±13.4) ng/g. A further decrease in cortisol was observed at the end of 2 h to an average of 11.5 (±6.7) ng/g (Table 1).

### 3.3 Experiment 2: Flow Treatments

At the end of the study, mean weight, FCR and survival did not differ significantly among the three flow treatments. Mean (±SD) golden shiner weights were 1.85 ± 0.10, 1.73 ± 0.24, and 2.07 ± 0.27 g, respectively for the 1, 2, or 4 cm/s treatment groups (Table 1). Survival ranged from 47 to 69% (Table 1). The observed FCR was between 1.49 and 1.83 (Table 1). On average, fish in the three treatment groups gained 52% of the initial body weight (1.24 g) during the 28-day period.

Whole-body cortisol measurements were not significantly different among the three flow treatments. Cortisol measurements, however, varied significantly across time intervals (F_4, 60_ = 12.67; *p* < 0.001). The observed baseline cortisol was on average 3.9 ± 1.4 ng/g body weight. A significant increase in mean cortisol was observed 30 min after the acute challenge. Cortisol concentrations remained elevated at the end of 2 h (71.7 ± 41.60 ng/g) in all the treatment groups (Table 1).

### 3.4 Necropsy

Most dead fish in Experiment 2 showed external clinical signs of columnaris disease such as saddlebacks, yellow abdomens, and reddened gills. On examination of the BHI and Hsu Shots plates after 48 h, bacterial growth was observed. The Hsu-Shots medium is used specifically to promote *F. covae* growth, the causative agent of columnaris disease confirming that some if not all fish in the experimental set-up harbored the bacterium.

## 4 Discussion

Laboratory experiments were conducted to evaluate crowding and water flow effects on golden shiner growth and physiology. The results and conclusions from the study are limited to raising fish in the laboratory and may not reflect actual commercial practices in TEP or SPS.

Growth was slow during both experiments but was comparable to previous indoor studies conducted by Lochmann et al. (2001) and Melandri et al. (2008). The slow growth in indoor laboratory studies is frequently attributed to the lack of natural foods in the experimental system (Lochmann and Phillips 1996). Crowding was an additional factor that might have contributed to the slow growth. The lowest stocking density (600 fish/m^3^) was used to model “confinement” in the SPS. Fish in the lowest density corresponded to 375,000 fish/ha but in a SPS they would be crowded into 20% of the total surface area resulting in an effective stocking density of 1,875,000 fish/ha. Golden shiners are a shoaling species (Pitcher 1983) and can “tolerate” crowding, but higher densities negatively influence individual golden shiner size and growth (Stone et al., 2016). Zebrafish, which is also a social and a shoaling species are known to display agonistic behaviors, especially when establishing dominance hierarchies in laboratory settings (Harper and Lawrence 2016). However, such agonistic behaviors were not observed in the golden shiners used in this study.

The weight gain during the flow study was slightly better (52% increase) than the crowding study (20% increase). This could have possibly been due to the positive effects of sustained swimming in a current of water. Many studies have suggested that sustained swimming in low to moderate currents of water can result in improved growth and feed utilization in many fishes (Davison 1997; Brown et al., 2011). In a SPS, there is a slow movement of water (∼2 cm/s), which could benefit growth or at the very least show no adverse effects (Stone and Rowan 1998). Likewise, data obtained for FCR revealed similar patterns. Although the fish were fed a high protein (42%) diet, the FCR was higher than those observed in pond studies (FCR = 0.9; Lochmann et al., 2004). FCR in the flow study was slightly lower (better) than the crowding study.

Survival in the flow study was between 47 and 69% which was much lower compared to the crowding study (91–98%). Fish in the flow study had succumbed to columnaris. This was likely a result of holding fish in a tank until the crowding study was completed. Holding fish for prolonged periods in tanks likely resulted in chronic stress making them vulnerable to disease outbreaks such as columnaris. *F. covae* is ubiquitous in aquatic environments and behaves as a secondary pathogen; stress-mediated pathogenesis is not uncommon (Mohammed 2015).

Cortisol is the primary glucocorticoid hormone released when teleost fish are subject to stress; its circulating levels in the bloodstream vary depending on the nature, intensity, and duration of the stressor (Mommsen et al., 1999). It should be noted that MS-222 does not affect cortisol concentrations when fish are euthanized (Holloway et al., 2004, Studies eliciting stress response after an acute challenge indicate that cortisol increases rapidly to reach a peak within an hour and then subsides after a few hours (Iwama et al., 2006). Likewise, in golden shiners, plasma cortisol peaked 2 h after acute crowding stress and subsided 2 h later (Lochmann et al., 2003). In small fish when phlebotomy does not yield an adequate volume of sample for measurement, several samples must be pooled, which can make results unreliable (Lochmann et al., 2003). Whole-body cortisol measures are used for small-sized fish such as golden shiners (Sink et al., 2007) and zebrafish (Ramsay et al., 2006). The differences in eliciting the stress response in the various studies make it difficult to draw comparisons.

In the present study, whole-body cortisol was used to measure chronic and acute stress in golden shiners. In both experiments, baseline cortisol was within the range of those obtained for golden shiners under non-stressful conditions (Melandri et al., 2008; Sink and Lochmann 2008). Interestingly, the baseline cortisol was similar in both experiments. However, the “normal” baseline cortisol (in Experiment 2), does not quite reflect the consequent responses to the acute challenge or the contraction of columnaris. While fish in the crowding study recovered within the 2 h observation period after the 1-min acute challenge, cortisol remained elevated in the flow study. The observations suggest that although the fish in the flow study had become accustomed to the chronic stress, their ability to respond to further acute challenges had become compromised. Consequently, the fish needed a longer recovery time after the acute stress challenge. The hidden costs of chronic stress over prolonged periods were emphasized by the poor physiological and pathophysiological responses.

Similar observations have been noted by Pickering and Stewart (1984), and Haukenes et al. (2004) where fish can acclimate to chronic stressors leading to a similar cortisol response among the various treatments. It is not likely that golden shiners are stressed greater by flow rate compared to crowding, rather the response could have been due to the prolonged holding in the flow-through tank prior to the initiation of the flow experiment. The additive effect of the flow, additional holding period (compared to fish in the crowding study) and the columnaris stressors could also have effected the whole-body cortisol results. The results from the crowding study might have been different for a longer study period; or for that matter, had the crowding study been conducted after the flow study. The 28-days period may not have been sufficient to elicit the long-term effects from the allostatic load (the cost that accelerates disease processes (McEwen 2000)) (Schreck 2000).

## 5 Conclusion

Even though, golden shiners are naturally a shoaling species of fish (Pitcher 1983) and could tolerate the tested densities and water circulation (Stone and Rowan 1998), long-term and compounded effects of the two warrant further research. Considering an annual production cycle, fish are likely to spend at least 180–200 days (if not more) between their juvenile and saleable periods. It is therefore required to carry out studies for longer durations to model a commercial scenario. While fish can become acclimated to chronic stressors and show no external signs of stress, the effects of the allostatic load may be reflected in subsequent acute challenges such as handling or transportation. Interestingly, in TEP, farmers manipulate preferred market sizes of baitfish by varying fish density. Therefore, it can be assumed that golden shiners can tolerate some crowding in the confined area of a SPS. However, it may be a challenge to maintain the health of fish for long periods under varying environmental conditions. *F. columnare* is a ubiquitous pathogen in not only aquatic environments including aquaculture ponds (Durborow et al., 1998), but also, is a part of the natural microbiome of freshwater fish (Barker et al., 1990). Hence it becomes necessary to investigate crowding and water flow effects on health in addition to growth.

## Data Availability

The raw data supporting the conclusions of this article will be made available by the authors, without undue reservation.
